# An Introduction to Practical Pharmacy: Designed as a Text-Book for the Student, and as a Guide to the Physician and Pharmaceutist, with Many Formulas and Prescriptions

**Published:** 1856-01

**Authors:** 


### BOOK NOTICES.

*An Introduction to Practical Pharmacy:* Designed as a Text-Book for the Student, and as a guide to the Physician and Pharmaceutist, with many Formulas and Prescriptions. By EDWARD PARRISH, graduate in Pharmacy, &c., &c.; with two hundred and forty-three illustrations. Philadelphia: Blanchard & Lea.

We are glad to receive this excellent work. It will supply a want long felt by the profession, and especially by the student of Pharmacy. A large majority of physicians are obliged to compound their own medicines, and to them a work of this kind is almost indispensible.

The volume is divided into parts, the contents of which will be understood from the following extract from the preface:

"In part I. are grouped several chapters of a preliminary character, among which, metrology, including weights and measures, and specific gravity, holds a prominent place; it is treated with an effort at simplicity, which should attract the student to its careful study.

Galenical Pharmacy occupies Part II.; the mode of preparing each of the various classes of permanent vegetable preparations prefaces a tabular statement of the relative strength, doses, and relative properties of the individual members.

This compact form of displaying the leading facts of the subject will be observed as a conspicuous feature of the work, and is designed to adapt it particularly to the use of the student. For making the officinal preparations, distinct and definite formulas are omitted, being given in the *Pharmacopœia*, which, as now published in a cheap form, it is presumed every physician and apothecary will possess and use. Unofficinal preparations are treated of more in detail, and hence occupy relatively a larger space. The order in which the preparations are introduced, is that which experience in the 'School of Practical Pharmacy' has indicated as best for the student; those most easily prepared are first treated of, and by gradations the more complex are brought forward; the whole arrangement of Galenical preparations being thus founded primarily upon the several processes of pulverization, solution, maceration, displacement, evaporation, and distillation, and secondarily upon the menstrua used in making them, their medical properties and uses.

Part III. is devoted to the classification of plants, giving in extensive syllabi almost all the leading articles of the materia medica, arranged on the basis of chemical composition.

The vague and uncertain analysis of many plants, and parts of plants, and our ignorance in regard to the real composition of many of their active principles, takes from this part of the work much of the value it would otherwise possess. Advantage is taken of these headings to introduce a variety of secondary organic products of great interest and importance, among which are the entire class of vegetable acids and alkaloids.

In part IV. the essential facts in regard to the inorganic medicines are briefly stated, and shown also in syllabi.

Part V. contains practical directions for prescribing, selecting, combining and dispensing medicines, illustrated by a considerable number of formulas or prescriptions variously written in Latih and English, abbreviated and unabbreviated. The attention of physicians is asked to this part of the work as showing many of the best modes of prescribing many of the more important drugs ; it will be observed that in the selection of prescriptions for publication in this connection, I have availed myself of the skill of numerous practitioners of medicine, some of whom are well known, besides introducing many standard extemporaneous preparations which the physician often finds occasion to prescribe, and the pharmaceutist to prepare and dispense.

The work is certainly a very valuable addition to our professional literature, and we commend it to the favorable consideration of both physicians and students.

For sale by Keen & Lee. J.

